# Apigenin inhibits osteoblastogenesis and osteoclastogenesis and prevents bone loss in ovariectomized mice

**DOI:** 10.1007/s10616-014-9694-3

**Published:** 2014-02-06

**Authors:** Tadashi Goto, Keitaro Hagiwara, Nobuaki Shirai, Kaoru Yoshida, Hiromi Hagiwara

**Affiliations:** 1 Department of Biomedical Engineering, Toin University of Yokohama, 1614 Kurogane-cho, Aoba-ku, Yokohama, 225-8503 Japan; 2 Department of Biological Sciences, Tokyo Institute of Technology, 4259 Nagatsuta-cho, Midori-ku, Yokohama, 226-8501 Japan; 3 Division of Molecular and Cellular Medicine, National Cancer Center Research Institute, 5-1-1, Tsukiji, Chuo-ku, Tokyo, 104-0045 Japan; 4 Tsukuba Laboratories, Nemoto Science Co. Ltd, 6136-4 Ohnogoh-machi, Joso-shi, Ibaraki 300-2521 Japan; 5 Biomedical Engineering Center, Toin University of Yokohama, 1614 Kurogane-cho, Aoba-ku, Yokohama, 225-8503 Japan

**Keywords:** Apigenin, Polyphenol, Osteoblast, Osteoclast, Ovariectomized mice, Bone loss

## Abstract

Polyphenol have been reported to have physiological effects with respect to alleviating diseases such as osteoporosis and osteopetrosis. We recently reported that the olive polyphenol hydroxytyrosol accelerates bone formation both in vivo and in vitro. The present study was designed to evaluate the in vivo and in vitro effects of apigenin (4′,5,7-trihydroxyflavone), one of the major polyphenols in olives and parsley, on bone formation by using cultured osteoblasts and osteoclasts and ovariectomized (OVX) mice, respectively. Apigenin markedly inhibited cell proliferation and indices of osteoblast differentiation, such as collagen production, alkaline phosphatase activity, and calcium deposition in osteoblastic MC3T3-E1 cells at concentrations of 1–10 μM. At 10 μM, apigenin completely inhibited the formation of multinucleated osteoclasts from mouse splenic cells. Moreover, injection of apigenin at 10 mg kg^−1^ body weight significantly suppressed trabecular bone loss in the femurs of OVX mice. Our findings indicate that apigenin may have critical effects on bone maintenance in vivo.

## Introduction

In bone tissues, both the formation and maintenance of bone are controlled by bone-forming osteoblasts and bone-resorbing osteoclasts, and an imbalance between these two cell types leads to bone metabolic diseases such as osteoporosis and osteopetrosis (Riggs 1987). Bone formation involves a complex series of events that include the proliferation and differentiation of osteoprogenitor cells, resulting in the formation of a mineralized extracellular matrix. The deposition of calcium and the sequential expression of type I collagen, alkaline phosphatase, and osteocalcin are known as markers of osteoblastic differentiation. Several model systems have been developed for studying the proliferation and differentiation of bone-forming cells in vitro and the molecular biology of the mineralization process, such as preosteoblastic cells from mouse calvariae (MC3T3-E1 cells) and osteoblast-like cells from rat calvariae (Bredford et al. 1993; Hagiwara et al. 1996; Liu et al. 1994; Stein et al. 1990). Osteoclasts are multinucleated giant cells with the ability to resorb mineralized tissues. They are formed from hematopoietic cells of the monocyte/macrophage lineage (Udagawa et al. 1990). The development of osteoclasts in culture is strictly dependent on support provided by osteoblasts and/or stromal cells (Udagawa et al. 1990). The formation and activation of osteoclasts are controlled by the combined actions of receptor activator of nuclear factor-κB ligand (RANKL) and macrophage colony-stimulating factor (M-CSF). Here, we used a single culture of mouse spleen cells with added M-CSF and soluble RANKL (Notoya et al. 2007).

**Fig. 1:**
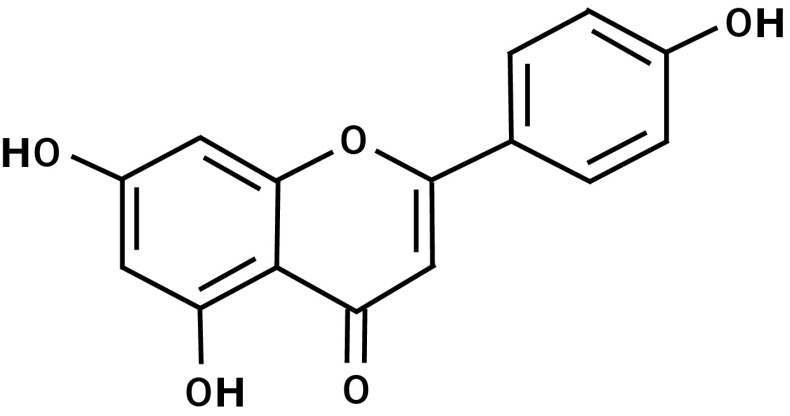
Structure of apigenin

Apigenin (4′,5,7-trihydroxyflavone), a member of the flavone family of flavonoid compounds (Fig. 1), is widely distributed in many vegetables and fruits including olives, parsley, and apples. This compound has antitumor and antioxidant properties (Patel et al. 2007; Shukla and Gupta 2010). In addition, apigenin has been found to have significant effects on inhibiting growth, arresting the cell cycle, and inducing apoptosis in many cancers and leukemia (Budhraja et al. 2012; Shukla and Gupta 2007; Zhu et al. 2013). Apigenin is also known to be a strong inhibitor of ornithine decarboxylase activity (Wei et al. 1990), LPS-induced cyclooxygenase-2 and nitric oxide synthase-2 activity (Liang et al. 1999), and protein kinase C activity (Lee and Lin 1997). A few studies have reported that apigenin inhibits osteoclastogenesis and osteoclast function (Bandyopadhyay et al. 2006). However, little information is available on the effects of apigenin on bone metabolism.

The present study was designed to evaluate the in vivo and in vitro effects of apigenin on the formation and maintenance of bone by using mice and cultured mouse cells, respectively. We also investigated preventive effects of apigenin on bone loss in adult mice with ovariectomy-induced osteoporosis. Our results indicate that apigenin may be useful in the prevention and treatment of osteoporosis.

## Materials and methods

### Materials

Apigenin was purchased from Enzo Life Science (Plymouth, PA, USA). α-Modified minimum essential medium (α-MEM), RPMI 1640 medium, and penicillin/streptomycin antibiotic mixture were obtained from Life Technologies, Inc. (Grand Island, NY, USA). Fetal bovine serum was obtained from Moregate BioTech (Bulimba, Australia). Recombinant murine M-CSF and recombinant human soluble RANKL (sRANKL) were from R&D Systems (Minneapolis, MN, USA) and Pepro Tech EC., Ltd. (London, UK), respectively.

### Osteoblastic cell cultures

Preosteoblastic MC3T3-E1 cells were obtained from RIKEN Cell Bank (Tsukuba, Japan). Cells were maintained in a 55-cm^2^ dish in α-MEM, supplemented with 10 % fetal bovine serum, 50 units mL^−1^ penicillin and 50 μg mL^−1^ streptomycin, in a humidified atmosphere of 5 % CO_2_ in air at 37 °C. After reaching 70 % confluence, cells were detached by treatment with 0.05 % trypsin, replated in either 55-cm^2^ dishes or 12-well plates (area of each well, 3.8 cm^2^) at a density of 1 × 10^4^ cells cm^−2^, and grown in α-MEM supplemented with 10 % fetal bovine serum, 50 units mL^−1^ penicillin, 50 μg mL^−1^ streptomycin, 5 mM β-glycerophosphate (Sigma-Aldrich, Tokyo, Japan), and 50 μg mL^−1^ ascorbic acid (Sigma-Aldrich). Fresh medium and apigenin were supplied to cells at 2-day intervals. Apigenin at 1, 5, and 10 μM was added to medium according to previous study (Bandyopadhyay et al. 2006). MC3T3-E1 cells formed nodules, and mineralization of nodules was observed after cultivation for 2–3 weeks.

### Formation of osteoclastic cells

Multinucleated osteoclastic cells formed from spleen cells by adding osteoclast differentiating factors, RANKL and M-CSF. Spleen cells were collected from the splenic tissues of 6-week-old male ddY mice (Sankyo Laboservice, Tokyo, Japan). Erythrocytes contaminating the spleen cell fraction were eliminated by adding 0.83 % ammonium chloride in 10 mM Tris–HCl (pH 7.4) to the cell pellet. Mouse spleen cells (2.4 × 10^5^ cells/well) in 96-well plates (0.32 cm^2^/well) were cultured with 50 ng mL^−1^ human sRANKL and 30 ng mL^−1^ M-CSF for 7 days. Cultures were maintained at 37 °C in a humidified atmosphere of 5 % CO_2_ in air. Fresh medium and apigenin at 1, 5, 10 μM were supplied at 2-day intervals. Multinucleated osteoclastic cells formed were fixed on the well in 3.7 % formaldehyde for 5 min and then in a mixture of ethanol and acetone (1:1; v:v) for 1 min. These were then stained for tartrate resistant acid phosphatase (TRAP) activity, one of osteoclastic differentiate markers. TRAP-positive multinucleated cells (five or more nuclei) were counted under a microscope (IX70; Olympus, Tokyo, Japan).

The Institutional Animal Care and Use Committee of Toin University of Yokohama approved all animal protocols and procedures.

### Toxicity of apigenin for cells

MC3T3-E1 cells were replated in 96-well plates (area of each well, 0.32 cm^2^) at a density of 1 × 10^3^ cells cm^−2^ and grown in α-MEM supplemented with 10 % fetal bovine serum, 50 units mL^−1^ penicillin, 50 μg mL^−1^ streptomycin, and apigenin at various concentrations. After subculture for 53 or 74 h, the cell layers were washed with RPMI 1640 medium. 3-[4,5-Dimethylthiazol-2-yl]-2,5-diphenyltetrazolium bromide (MTT; DOJINDO, Kumamoto, Japan) reagent (0.5 mg mL^−1^ RPMI 1640) was added to each well, followed by incubation for 4 h for formazan formation. After the medium was removed, dimethyl sulfoxide was added to each well to dissolve the formazan, and absorbance was measured at 570 nm. The absorbance of the solution at 570 nm was related to the number of live cells.

### Sirius Red staining of collagen

Collagen was stained with Sirius Red (Tullberg-Reinert and Jundt, 1999). Sirius Red F3BA was purchased from Polysciences, Inc. (Wamington, PA, USA). Dye was dissolved in saturated aqueous picric acid at a concentration of 100 mg dL^−1^. Cells were plated in 24-well plates (2.0 cm^2^/well) at a density of 1 × 10^4^ cells cm^−2^ and subcultured with apigenin for 3 days. Cell layers were air-dried overnight on a sterile bench and fixed with 1 mL of Bouin’s solution (Sigma, St. Louis, MO, USA) for 1 h. Fixation fluid was removed by suction and culture plates were washed by immersion in running tap water for 15 min. Culture plates were air-dried, and 1 mL of Sirius Red dye reagent was added. Cells were stained for 1 h with shaking on a plate shaker. Stained cell layers were washed with 0.01 N hydrochloric acid to remove all non-bound dye, and stained material was dissolved in 0.5 mL of 0.1 N sodium hydroxide by using a plate shaker for 30 min at room temperature, after which the absorbance was measured at 550 nm.

### Measurement of alkaline phosphatase activity

MC3T3-E1 cells were subcultured in 12-well plates (3.8 cm^2^/well) in α-MEM containing 10 % fetal bovine serum, 5 mM β-glycerophosphate, and 50 μg mL^−1^ ascorbic acid. After the cells had reached confluence (day 3), apigenin was added to cultures at various concentrations for 12 days. Cells were washed with 10 mM Tris–HCl, pH 7.2, and were sonicated in 1 mL of 50 mM Tris–HCl (pH 7.2) containing 0.1 % Triton X-100 and 2 mM MgCl_2_ for 15 s with a sonicator (Ultrasonic Disruptor UD-201; Tomy Co., Tokyo, Japan). Alkaline phosphatase activity was determined using an established technique with *p*-nitrophenyl phosphate as the substrate (Hagiwara et al. 2011). Protein concentrations were determined using BCA protein assay reagent (Pierce Chemical Co., Rockford, IL, USA) with bovine serum albumin as a standard.

### Quantitation of calcium deposition

MC3T3-E1 cells were subcultured in α-MEM containing 10 % fetal bovine serum, 5 mM β-glycerophosphate, and 50 μg mL^−1^ ascorbic acid. After the cells had reached confluence (day 3), apigenin was added at various concentrations to the culture medium and cells were subcultured for 15 days. The amount of calcium, deposited as hydroxyapatite in the cell layer, was measured as follows: Layers of cells in 12-well plates (3.8 cm^2^/well) were washed with PBS and incubated overnight with 1 mL of 2 N HCl with gentle shaking. Ca^2+^ ions in the samples were quantitated by the *o*-cresolphthalein complexone method with a Calcium C kit (Wako Pure Chemical Industries, Osaka, Japan). This kit is specific for Ca^2+^ ions and has a detection limit of 1 μg mL^−1^. The solution of Ca^2+^ ions (20 mg dL^−1^) provided in the kit was used as the standard solution.

### Analysis of bone mineral density of OVX mice treated with apigenin

BALB/c female mice (6-weeks old) were purchased from Sankyo Laboservice (Tokyo, Japan) and were housed individually at 24 °C with a 12 h light–dark cycle. The mice underwent a sham-operation (n = 5) or were surgically ovariectomized (OVX; n = 10) under anesthesia with Nembutal^®^ injection (Dainippon Sumitomo Pharma, Tokyo, Japan). The mice were assigned to three groups (n = 5 for each): (1) untreated (Sham: sham-operated controls); (2) untreated (OVX controls); and (3) OVX administered intraperitoneally with apigenin (10 mg kg^−1^ body weight) at 2-day intervals. Apigenin was dissolved in ethanol and was diluted tenfold with saline before injection. The volume ingested was 50 μL. After the 28-day experimental period, the left and right femurs were surgically obtained from the anesthetized mice. The success of ovariectomy was confirmed by uterine atrophy in OVX mice.

The bone mineral density of the femurs was assessed using an X-ray CT System (LA Theata LCT-100; Aloka, Tokyo, Japan). We monitored the bone mineral density of femurs at 0.3 mm intervals and separately analyzed each trabecular and cortical bone. Values at 2.1 and 2.4 mm from the epiphysis of the femur are shown in the figures. Animal protocols and procedures were approved by the Institutional Animal Care and Use Committee of the Toin University of Yokohama.

### Statistical analysis

Numerical data have been expressed as mean ± SD values of the results from three to four cultures, and the significance of differences was analyzed by ANOVA (Dunnett’s test). Statistical significance was set at *P* < 0.05. Experiments were repeated independently in triplicate and the results were qualitatively identical in every case. Results from representative experiments are shown.

## Results

### Effects of apigenin on cultured osteoblasts

**Fig. 2:**
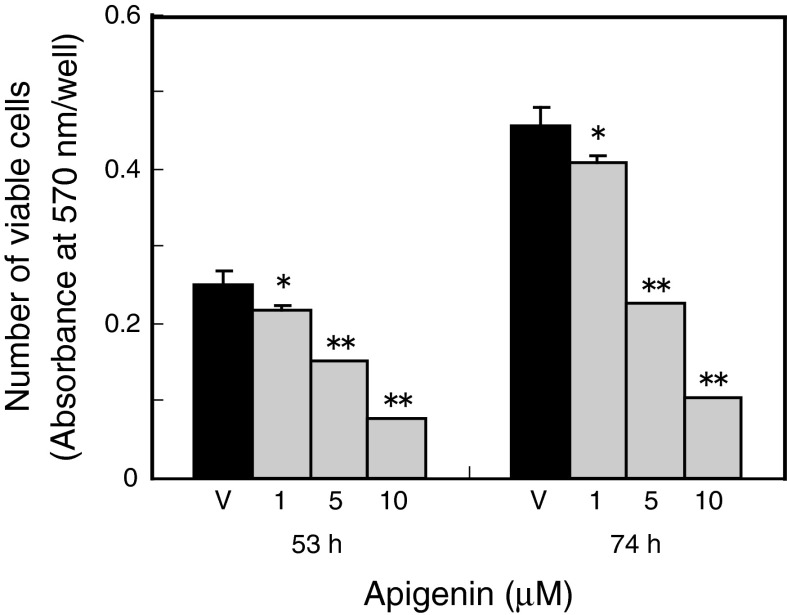
Effects of apigenin on osteoblastic cell viability. MC3T3-E1 cells (1 × 10^3^ cells/well; 96-well plates) were exposed to the olive polyphenol apigenin at various concentrations (1–10 μM) and were subcultured for 53 or 74 h. After treatment with apigenin, the cells were treated with MTT (50 μg/well) for 4 h, and the absorbance at 570 nm was measured. The values represent the mean ± SD of results from three wells. Data are representative of results from three separate experiments. *P < 0.05 versus vehicle (V) and **P < 0.01 versus vehicle (V)

We first evaluated the toxicity of apigenin for MC3T3-E1 cells by using the MTT assay. Apigenin decreased in the number of viable MC3T3-E1 cells in a dose-dependent manner (Fig. 2). Exposure of MC3T3-E1 cells to 10 μM apigenin decreased in viable cell number by approximately 70 and 77 % at 53 and 74 h, respectively, relative to control cultures treated with the vehicle alone. However, exposure of MC3T3-E1 cells to apigenin at 10 μM did not affect cell morphological features.

**Fig. 3:**
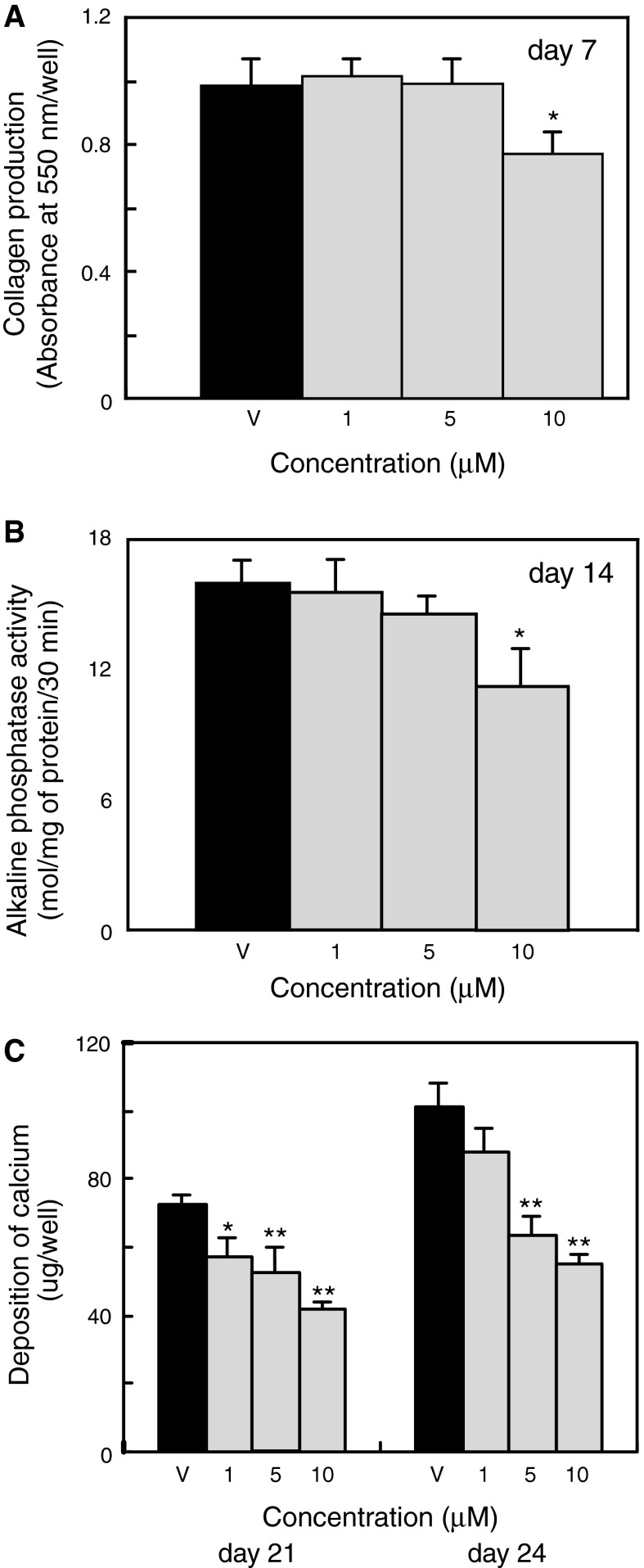
Effects of apigenin on collagen production, alkaline phosphatase activity, and mineralization of osteoblasts. MC3T3-E1 cells were cultured in 12-well plates (3.8 cm^2^/well) or 24-well plates (2.0 cm^2^/well) with α-MEM containing 10 % fetal bovine serum, 5 mM β-glycerophosphate, and 50 μg mL^−1^ ascorbic acid. After the cells reached confluence (day 3), the olive polyphenol apigenin was added at various concentrations (1–10 μM) to the culture medium. Fresh medium with test compound was supplied at 3-day intervals. **a** Collagen production was measured at day 7 as described in the method. Cell layers in 24-well plates (2.0 cm^2^/well) were fixed with Bouin’s solution and stained with Sirius Red dye reagent. Stained material was dissolved in 0.1 N sodium hydroxide and the absorbance was measured at 550 nm. **b** Alkaline phosphatase activity was measured at day 14 as described in the method. **c** Deposition of Ca^2+^ ions was measured at days 21 and 24. Quantitative analysis of Ca^2+^ ions was performed as described in the method. All values represent the mean ± SD of the results from three wells. Data are representative of results from three separate experiments. *P < 0.05 versus vehicle (V) and **P < 0.01 versus vehicle (V)

To assess the effects of apigenin on the differentiation and mineralization of MC3T3-E1 cells, we added apigenin to the culture medium of post-proliferative cells and assayed collagen production, alkaline phosphatase activity, and calcium deposition (Fig. 3). Collagen is an early stage marker of osteoblastic differentiation, and as shown in Fig. 3a, incubation of cells with 10 μM apigenin for 7 days significantly inhibited the production of collagen. We then examined the activity of alkaline phosphatase, a middle-stage marker of osteoblastic differentiation, in osteoblastic cells. Apigenin significantly decreased the activity of alkaline phosphatase in MC3T3-E1 cells on day 14 when used at 10 μM (Fig. 3b). Furthermore, as demonstrated in Fig. 3c, apigenin dose-dependently inhibited calcium deposition by MC3T3-E1 cells on days 21 and 24. Exposure of MC3T3-E1 cells to 10 μM apigenin decreased calcium deposition by approximately 43 and 45 % on days 21 and 24, respectively, relative to control cultures treated with the vehicle alone (Fig. 3c).

### Effects of apigenin on the formation of multinucleated osteoclasts

**Fig. 4:**
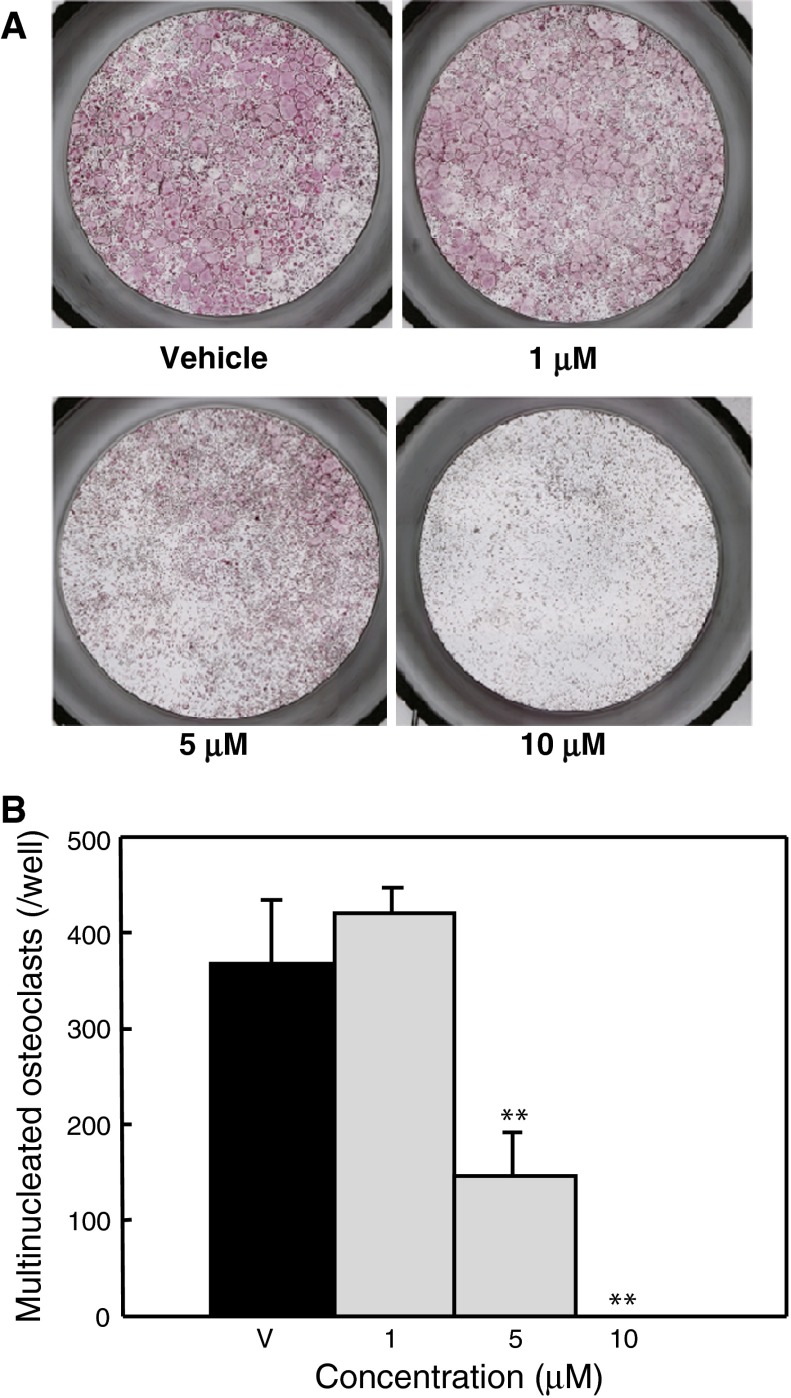
Effects of apigenin on osteoclast formation. Osteoclasts were formed from mouse spleen cells. **a** Typical results of staining for the detection of TRAP activity. Apigenin was added to cultures at the indicated concentrations. Cultured cells were then stained for TRAP activity on day 7. **b** Apigenin was added to cultures at the indicated concentrations. Cultured cells were then stained for TRAP activity on day 7. TRAP-positive multinucleated cells (five or more nuclei) were counted under a microscope. *Columns and bars* show mean ± SD values of the results from five wells. Data are representative of the results of three separate experiments. **P < 0.01 versus vehicle (V)

Multinucleated osteoclastic cells were formed from mouse splenic cells by the addition of 30 ng mL^−1^ M-CSF and 50 ng mL^−1^ human recombinant sRANKL. Figure 4a shows representative results for the detection of TRAP activity in multinucleated osteoclastic cells treated with apigenin at the indicated concentrations in the figure. Formation of TRAP-positive multinucleated osteoclastic cells was dose-dependently inhibited by the addition of apigenin (Fig. 4b). Exposure of splenic cells to 10 μM apigenin completely inhibited the formation of multinucleated osteoclastic cells relative to control cultures treated with the vehicle alone.

### Effects of polyphenols on bone loss in OVX mice

**Fig. 5:**
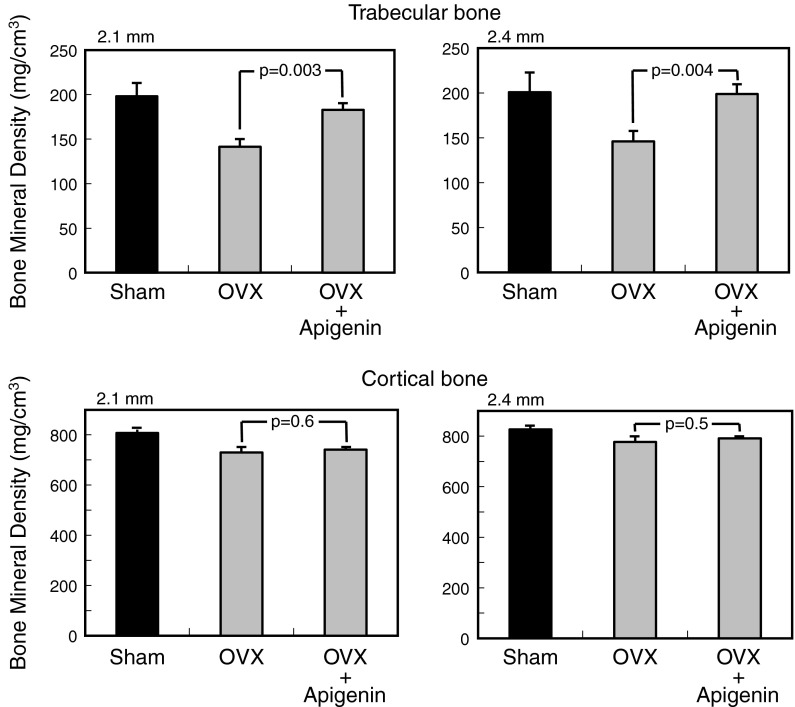
Effects of apigenin on the bone mineral density of the femurs of OVX mice. BALB/c female mice (6-week old) underwent a sham operation (n = 5) or were surgically ovariectomized (OVX; n = 10) under anesthesia. Mice were assigned to three groups (n = 5 for each): Sham, sham-operated controls; OVX controls; OVX + Api, OVX administered intraperitoneally with apigenin (10 mg kg^−1^ body weight) at 3-day intervals. After the 28-day experimental period, the left and right femurs were surgically collected from anaesthetized mice. Bone mineral density of the femurs (trabecular bone and cortical bone) was assessed using an X-ray CT System (LA Theata LCT-100; Aloka). It was monitored at 0.3 mm intervals, and the values at 2.1 mm and 2.4 mm from the femur epiphysis are shown in the figures

We examined the effects of apigenin on bone mineral density by using OVX mice. We used a polyphenol dose of 10 mg kg^−1^ body weight because this concentration of polyphenol has been reported to be effective in vivo in previous studies (Hagiwara et al. 2011; Notoya et al. 2006). Compared with the values for the Sham group (18.8 ± 1.9 g), the final body weights in the OVX group (20.8 ± 0.8 g) were significantly increased at 28 days after the operation. There were no differences in body weight between the OVX group (20.8 ± 0.8 g) and the OVX-administered apigenin group (21.0 ± 1.3 g) at 28 days after the operation. Ovariectomy induced a severe decrease in the bone mineral density of the trabecular bone of mice (Fig. 5). At 2.1 and 2.4 mm from the epiphysis of the femur, the bone mineral density in the OVX group was significantly lower than that in the Sham group (65 and 67 %, respectively). Administration of apigenin at 10 mg kg^−1^ body weight significantly reduced the loss of trabecular bone in OVX mice (Fig. 5). At 2.1 and 2.4 mm from the epiphysis of the femur, significant recovery (83 and 91 %, respectively) of bone mineral density was observed in the OVX-administered apigenin group compared to that in the OVX group. In contrast, apigenin did not affect the loss of cortical bone mineral density in OVX mice.

## Discussion

We screened natural polyphenols for the ability to regulate the proliferation, differentiation, and function of cultured osteoblasts and osteoclasts in order to identify factors that may cause, prevent, or treat bone metabolic diseases such as osteoporosis and osteopetrosis. We had previously reported that the isoflavone genistein attenuates osteoclastogenesis by decreasing the levels of receptor activator NF-κB ligand mRNA in osteogenic/stromal cells (Yamagishi et al. 2001). Quercetin (Notoya et al. 2004) and curcumin (Notoya et al. 2006) have been reported to inhibit cultured osteoblast metabolism. In addition, curcumin (Bharti et al. 2004) and quercetin (Woo et al. 2004) have been found to inhibit osteoclastogenesis. Kamon et al. (2010) reported that the green tea polyphenol epigallocatechin gallate inhibited the differentiation of murine osteoblastic MC3T3-E1 cells and the formation of osteoclasts. Recently, we showed that the olive polyphenols oleuropein and hydroxytyrosol accelerate osteoblast differentiation and mineralization, inhibit osteoclast formation in culture, and attenuate bone loss in OVX mice (Hagiwara et al. 2011). Thus, polyphenols regulate bone metabolism in culture via osteoblasts and osteoclasts.

In this study, we attempted to clarify the potential effects of apigenin on bone metabolism. We found that apigenin inhibited osteoblast differentiation markers such as type I collagen production, alkaline phosphatase activity, and calcium deposition by MC3T3-E1 osteoblasts. Furthermore, apigenin attenuated the formation of multinucleated osteoclasts in culture. It has also been reported that apigenin inhibits osteoclastogenesis and osteoclast function (Bandyopadhyay et al. 2006). These findings indicate that apigenin has inhibitory effects on both osteoblastogenesis and osteoclastogenesis in vitro. Therefore, to confirm whether apigenin contributes to reduction in the risk of osteoporosis, we investigated the preventive effects of apigenin on bone formation in vivo. Our results showed that apigenin markedly reduced bone loss in trabecular bone but had no effect on cortical bone loss in OVX mice. Experiments involving OVX female Sprague-Dawley rats (Park et al. 2008) have shown that apigenin prevents trabecular bone loss at a concentration of 10 mg kg^−1^ body weight. Thus, apigenin has been shown to reduce loss of bone mineral density in the trabecular bone in mice and rats.

It is well known that polyphenols have antioxidant properties (Rice-Evans et al. 1995). Recent reports have suggested that reactive oxygen species (ROS) play an important role in the regulation of cell proliferation, differentiation and metabolism. In particular, ROS inhibit the formation of bone by osteoblastic cells (Hosoya et al. 1998; Lee et al. 2006; Mody et al. 2001). Oxidative stress resulting in increased levels of intracellular ROS has been reported to suppress bone metabolism. Arai et al. (2007) reported that mineralization by MC3T3-E1 cells was reduced by half after a single exposure to H_2_O_2_ within the non-toxic concentration range. In addition, there have been some reports that H_2_O_2_ suppresses differentiation markers such as alkaline phosphatase activity, type I collagen gene expression, and the mineralization of osteoblastic cells (Hosoya et al. 1998; Lee et al. 2006; Mody et al. 2001). We had previously reported that apigenin does not demonstrate antioxidant effects in MC3T3-E1 cells (Hagiwara et al. 2011). Therefore, the inhibitory effects of apigenin on osteoblasts and osteoclasts in this study do not depend on a reduction in H_2_O_2_ levels.

In conclusion, the olive and parsley polyphenol apigenin markedly inhibited the formation of multinucleated osteoclasts in culture and prevented bone loss in an experimental model of osteoporosis (OVX mice). These findings suggest that apigenin may provide insights into the development of tools useful for the prevention and treatment of osteoporosis. Further investigation is required to clarify the detailed molecular mechanisms underlying the activity of apigenin in bone.

## References

[CR1] Arai M, Shibata Y, Pugdee K, Abiko Y, Ogata Y (2007). Effects of reactive oxygen species (ROS) on antioxidant system and osteoblastic differentiation in MC3T3-E1 cells. IUBMB Life.

[CR2] Bandyopadhyay S, Lion J-M, Mentaverri R, Ricupero DA, Kamel S, Romero JR, Chattopadhyay N (2006). Attenuation of osteoclastogenesis and osteoclast function by apigenin. Biochem Pharmacol.

[CR3] Bharti AC, Takada Y, Aggarwal BB (2004). Curcumin (diferuloymethane) inhibits receptor activator of NF-κB ligand-induced NF-κB activation in osteoclast precursors and suppresses osteoclastogenesis. J Immunol.

[CR4] Bredford JN, Graves SE, Smoothy CA (1993). Formation of mineralized nodules by bone derived cells in vitro: a model of bone formation?. Am J Med Genet.

[CR5] Budhraja A, Gao N, Zhang Z, Son YO, Cheng S, Wang X, Ding S, Hitron A, Chen G, Luo J, Shi X (2012). Apigenin induces apoptosis in human leukemia cells and exhibits anti-leukemic activity in vivo. Mol Cancer Ther.

[CR6] Hagiwara H, Inoue A, Yamaguchi A, Yokose S, Furuya M, Tanaka S, Hirose S (1996). cGMP produced in response to ANP and CNP regulates proliferation and differentiation of osteoblastic cells. Am J Physiol.

[CR7] Hagiwara K, Goto T, Araki M, Miyazaki H, Hagiwara H (2011). Olive polyphenol hydroxytyrosol prevents bone loss. Eur J Pharmacol.

[CR8] Hosoya S, Suzuki H, Yamamoto M, Kobayashi K, Abiko Y (1998). Alkaline phosphatase and type I collagen gene expressions were reduced by hydroxyl radical-treated fibronectin substratum. Mol Genet Metab.

[CR9] Kamon M, Zhao R, Sakamoto K (2010). Green tea polyphenol (-)-epigallocatechin gallate suppressed the differentiation of murine osteoblastic MC3T3-E1 cells. Cell Biol Int.

[CR10] Lee SF, Lin JK (1997). Inhibitory effects of phytopolyphenols on TPA-induced transformation, PKC activation, and c-jun expression in mouse fibroblast cells. Nutr Cancer.

[CR11] Lee DH, Lim BS, Lee YK, Yang HC (2006). Effects of hydrogen peroxide (H_2_O_2_) on alkaline phosphatase activity and matrix mineralization of odontoblast and osteoblast cell lines. Cell Biol Toxicol.

[CR12] Liang YC, Huang YT, Tsai SH, Lin-Shiau SY, Chen CF, Lin JK (1999). Suppression of inducible cyclooxygenase and inducible nitric oxide synthase by apigenin and related flavonoids in mouse macrophages. Carcinogenesis.

[CR13] Liu F, Malaval L, Gupta AK, Aubin JE (1994). Simultaneous detection of multiple bone-related mRNAs and protein expression during osteoblast differentiation: polymerase chain reaction and immunocytochemical studies at the single cell level. Dev Biol.

[CR14] Mody N, Parhami F, Sarafian TA, Demer LL (2001). Oxidative stress modulates osteoblastic differentiation of vascular and bone cells. Free Radic Biol Med.

[CR15] Notoya M, Tsukamoto Y, Nishimura H, Woo J-T, Nagai K, Lee I-S, Hagiwara H (2004). Quercetin, a flavonoid, inhibits the proliferation, differentiation, and mineralization of osteoblasts in vitro. Eur J Pharmacol.

[CR16] Notoya M, Nishimura H, Woo J-T, Nagai K, Ishihara Y, Hagiwara H (2006). Curcumin inhibits the proliferation and mineralization of cultured osteoblasts. Eur J Pharmacol.

[CR17] Notoya M, Arai R, Katafuchi T, Minamino N, Hagiwara H (2007). A novel member of the calcitonin gene-related peptide family, calcitonin receptor-stimulating peptide, inhibits the formation and activity of osteoclasts. Eur J Pharmacol.

[CR18] Park JA, Ha SK, Kang TH, Oh MS, Cho MH, Lee SY, Park J-H, Kim SY (2008). Protective effect of apigenin on ovariectomy-induced bone loss in rats. Life Sci.

[CR19] Patel D, Shukla S, Gupta S (2007). Apigenin and cancer chemoprevention: progress, potential and promise. Int J Oncol.

[CR20] Rice-Evans CA, Miller NJ, Bolwell PG, Bramley PM, Pridham JB (1995). The relative antioxidant activities of plant-derived polyphenolic flavonoids. Free Radic Res.

[CR21] Riggs BL (1987). Pathogenesis of osteoporosis. Am J Obstet Gynecol.

[CR22] Shukla S, Gupta S (2007). Apigenin-induced cell cycle arrest is mediated by modulation of MAPK, PI3 K-Akt, and loss of cyclin D1 associated retinoblastoma dephosphorylation in human prostate cancer cells. Cell Cycle.

[CR23] Shukla S, Gupta S (2010). Apigenin: a promising molecule for cancer prevention. Pharm Res.

[CR24] Stein GS, Lian JB, Owen TA (1990). Relationship of cell growth to the regulation of tissue-specific gene expression during osteoblast differentiation. FASEB J.

[CR25] Tullberg-Reinert H, Jundt G (1999). *In situ* measurement of collagen synthesis by human bone cells with a Sirius Red-based colorimetric microassay: effects of transforming growth factor β2 and ascorbic acid 2-phosphate. Histochem Cell Biol.

[CR26] Udagawa N, Takahashi N, Akatsu T, Tanaka H, Sasaki T, Nishihara T, Koga T, Martin TJ, Suda T (1990). Origin of osteoclasts: mature monocytes and macrophages are capable of differentiating into osteoclasts under a suitable microenvironment prepared by bone marrow-derived stromal cells. Proc Natl Acad Sci USA.

[CR27] Wei H, Tye L, Bresnick E, Birt DF (1990). Inhibitory effect of apigenin, a plant flavonoid, on epidermal ornithine decarboxylase and skin tumor promotion in mice. Cancer Res.

[CR28] Woo J-T, Nakagawa H, Notoya M, Yonezawa T, Udagawa N, Lee I-S, Ohnishi M, Hagiwara H, Nagai K (2004). Quercetin suppresses bone resorption by inhibiting the differentiation and activation of osteoclasts. Biol Pharm Bull.

[CR29] Yamagishi T, Otsuka E, Hagiwara H (2001). Reciprocal control of expression of mRNAs for osteoclast differentiation factor and OPG in osteogenic stromal cells by genistein: evidence for the involvement of topoisomerase II in osteoclastogenesis. Endocrinology.

[CR30] Zhu Y, Mao Y, Chen H, Lin Y, Hu Z, Wu J, Xu X, Xu X, Qin J, Xie L (2013). Apigenin promotes apoptosis, inhibits invasion and induces cell cycle arrest of T24 human bladder cancer cells. Cancer Cell Int.

